# Supplementary material for: Disseminated Intravascular Coagulopathy Is Associated with the Outcome of Persistent Inflammation, Immunosuppression and Catabolism Syndrome
Source: J Clin Med. 2020 Aug 17;9(8):2662. doi: 10.3390/jcm9082662 (PMC7464448; doi:10.3390/jcm9082662)
Supplement: Supplementary file 1 [file jcm-09-02662-s001.zip › supplementary table2.docx]

**Supplementary Table 2 Univariate/Multivariate Logistic Regression Analysis for persistent inflammation, immunosuppression and catabolism syndrome in patients with sepsis**

|  | **Univariate Logistic Regression Analysis** | |  | **Multivariate Logistic Regression Analysis** | |
| --- | --- | --- | --- | --- | --- |
| **PIICS** | **odds ratio (95% CI)** | ***p* value** |  | **odds ratio (95% CI)** | ***p* value** |
| **Age** | 1.01 (0.99–1.02) | 0.12 |  | 1.03 (1.01-1.05) | 0.010* |
| **Male** | 1.67 (1.21–2.29) | 0.0013* |  | 1.98 (1.16–3.40) | 0.011* |
| **SOFA** | 1.22 (1.16–1.29) | <0.0001* |  |  |  |
| **APACHEII** | 1.05 (1.04–1.08) | <0.0001* |  | 1.05 (1.02–1.08) | 0.0021* |
| **C reactive protein (mg/dl)** | 1.04 (1.02–1.06) | <0.0001* |  | 1.02 (0.99–1.04) | 0.26 |
| **Albumin (g/dl)** | 0.66 (0.56–0.77) | <0.0001* |  | 0.60 (0.38–0.93) | 0.020* |
| **Lymphocyte counts (x1000/μl) _#_** | 0.92 (0.77–1.10) | 0.34 |  | 1.02 (0.83-1.26) | 0.82 |
| **Hemoglobin (g/dl)** | 0.99 (0.94–1.04) | 0.68 |  | 1.13 (1.02-1.27) | 0.022* |
| **Creatinine (mg/dl)** | 1.12 (1.04–1.20) | 0.0046* |  | 1.05 (0.92-1.19) | 0.48 |
| **HbA1c (%)** | 1.11 (1.01–1.23) | 0.039 |  | 1.14 (0.98-1.33) | 0.092 |
| **ISTH overt DIC** | 1.83 (1.26–2.66) | 0.0019* |  | 1.22 (0.69–2.17) | 0.49 |
| **JAAM DIC** | 1.85 (1.32-2.59) | 0.0005* |  |  |  |
| **Antithrombin activity (%)** | 0.99 (0.98–0.99) | 0.021* |  | 1.00 (0.99-1.01) | 0.61 |

#; the unit is changed from usual one based on the clinical insights.

SOFA and JAAM DIC were excluded from the multivariable model since these variables had collinearity with APACHE and ISTH overt DIC, respectively.

PIICS; persistent inflammation, immunosuppression and catabolism syndrome, ISTH overt DIC; International Society on Thrombosis and Haemostasis overt disseminated intravascular coagulation, JAAM DIC; Japanese Association for Acute Medicine-disseminated intravascular coagulation, SOFA; sequential organ failure assessment, APACHE; acute physiology and chronic health evaluation
