# Supplementary material for: Disseminated Intravascular Coagulopathy Is Associated with the Outcome of Persistent Inflammation, Immunosuppression and Catabolism Syndrome
Source: J Clin Med. 2020 Aug 17;9(8):2662. doi: 10.3390/jcm9082662 (PMC7464448; doi:10.3390/jcm9082662)
Supplement: Supplementary file 1 [file jcm-09-02662-s001.zip › supplementary table3.docx]

**Supplementary table 3. Association between variables in the generalized structured equation modelling.**

| **Factor** | **Coefficient, (95%CI)** | **Standardized coefficient** | **P value** |
| --- | --- | --- | --- |
| **Severity APACHE2 score** |  |  |  |
| Age per year | 0.06 (0.05 to 0.07) | 0.14 | <0.001* |
| Male sex | -0.24 (-0.61 to 0.13) | -0.01 | 0.21 |
| Diabetes, HbA1c % | 3.03 (2.88 to 3.17) | 0.48 | <0.001* |
| (Correlation) Hemoglobin, g/dl | -4.39 (-4.85 to -3.94) | -0.22 | <0.001* |
| (Correlation) Infection | 0.06 (-0.01 to 0.14) | 0.02 | 0.11 |
| (Correlation) Renal dysfunction per creatinine, mg/dl | 1.76 (1.42 to 2.10) | 0.12 | <0.001* |
| **Renal dysfunction** **per creatinine, mg/dl** |  |  |  |
| Age per year | 0.01 (0.00 to 0.01) | 0.06 | <0.001* |
| Male sex | 0.53 (0.43 to 0.63) | 0.14 | <0.001* |
| Infection | 0.11 (-0.01 to 0.23) | 0.02 | 0.11 |
| Diabetes, HbA1c % | -0.06 (-0.10 to -0.02) | -0.04 | 0.002* |
| (Correlation) Hemoglobin, g/dl | -0.85 (-0.97 to -0.73) | -0.18 | <0.001* |
| (Correlation) Severity, APACHE2 score | 1.76 (1.42 to 2.10) | 0.12 | <0.001* |
| **Hemoblobin, g/dl** |  |  |  |
| Age per year | -0.03 (-0.03 to -0.02) | -0.19 | <0.001* |
| Male sex | 0.67 (0.54 to 0.80) | 0.13 | <0.001* |
| Infection | -0.35 (-0.50 to -0.19) | -0.06 | <0.001* |
| Diabetes, HbA1c % | 0.46 (0.41 to 0.51) | 0.23 | <0.001* |
| **Nutrition status, albumin, g/dL** |  |  |  |
| (Correlation) Inflammation, CRP, mg/dl | -1.85 (-2.01 to -1.70) | -0.27 | <0.001* |
| Infection | -0.41 (-0.46 to -0.36) | -0.19 | <0.001* |
| Severity, APACHE2 score | -0.02 (-0.02 to -0.02) | -0.17 | <0.001* |
| Renal dysfunction per creatinine, mg/dl | 0.01 (0.00 to 0.02) | 0.03 | 0.001* |
| Hemoglobin, g/dl | 0.15 (0.14 to 0.16) | 0.43 | 0.03 |
| **Inflammation, CRP, mg/dl** |  |  |  |
| Infection | 6.37 (5.91 to 6.83) | 0.34 | <0.001* |
| Severity, APACHE2 score | 0.03 (0.00 to 0.05) | 0.03 | 0.10 |
| Renal dysfunction per creatinine, mg/dl | 0.55 (0.44 to 0.65) | 0.13 | <0.001* |
| Hemoglobin, g/dl | -0.08 (-0.15 to 0.00) | -0.03 | 0.048* |
| (Correlation) Coagulopathy, ISTH overt DIC score | 0.08 (0.02 to 0.15) | 0.03 | 0.08 |
| (Correlation) Nutrition status, albumin, g/dl | -1.85 (-2.01 to -1.70) | -0.27 | <0.001* |
| **Coagulopathy, ISTH overt DIC score** |  |  |  |
| Infection | 0.04 (0.01 to 0.06) | 0.04 | 0.001* |
| Severity, APACHE2 score | 0.00 (0.00 to 0.01) | 0.11 | <0.001* |
| Renal dysfunction per creatinine, mg/dl | 0.01 (0.00 to 0.01) | 0.04 | 0.004* |
| Hemoglobin, g/dl | -0.02 (-0.02 to -0.01) | -0.11 | <0.001* |
| (Correlation) Inflammation, CRP, mg/dl | 0.08 (0.02 to 0.15) | 0.03 | 0.01* |
| **Others** |  |  |  |
| Age per year | 0.00 (0.00 to 0.00) | 0.03 | 0.004 |
| Male | 0.04 (0.00 to 0.09) | 0.02 | 0.073 |
| Infection | 0.34 (0.28 to 0.40) | 0.13 | <0.001* |
| Renal dysfunction per creatinine, mg/dl | 0.02 (0.01 to 0.04) | 0.04 | 0.001 |
| Severity, APACHE2 score | 0.04 (0.03 to 0.04) | 0.30 | <0.001* |
| Diabetes, HbA1c % | -0.12 (-0.14 to -0.10) | -0.15 | <0.001* |
| Hemoglobin g/dl | 0.03 (0.02 to 0.04) | 0.07 | <0.001* |
| **PIICS** |  |  |  |
| Inflammation, CRP, mg/dl | 0.01 (-0.01 to -0.01) | 0.17 | <0.001* |
| Coagulopathy, ISTH overt DIC score | 0.07 (-0.09 to -0.05) | 0.09 | <0.001* |
| Nutrition status, albumin, g/dL | -0.02 (0.01 to 0.03) | -0.06 | <0.001* |
| Others | 0.21 (0.20 to 0.22) | 0.75 | <0.001* |
| **Non-PIICS** |  |  |  |
| Inflammation, CRP, mg/dl | -0.01 (-0.01 to 0.00) | -0.11 | <0.001* |
| Coagulopathy, ISTH overt DIC score | -0.14 (-0.17 to -0.12) | -0.14 | <0.001* |
| Nutrition status, albumin, g/dL | 0.04 (0.02 to 0.05) | 0.09 | <0.001* |
| Others | -0.37 (-0.38 to -0.35) | -1.04 | <0.001* |

PIICS; persistent inflammation, immunosuppression and catabolism syndrome, ISTH DIC; International Society on Thrombosis and Haemostasis disseminated intravascular coagulation, APACHE; acute physiology and chronic health evaluation, CRP: C reactive protein, HbA1c; hemoglobin A1c
