# Supplementary material for: Disseminated Intravascular Coagulopathy Is Associated with the Outcome of Persistent Inflammation, Immunosuppression and Catabolism Syndrome
Source: J Clin Med. 2020 Aug 17;9(8):2662. doi: 10.3390/jcm9082662 (PMC7464448; doi:10.3390/jcm9082662)
Supplement: Supplementary file 1 [file jcm-09-02662-s001.zip › supplemantary table1.doc]

**Supplementary Table.1 Scoring system for disseminated intravascular coagulation**

|  | ISTH overt DIC criteria | JAAM DIC criteria |
| --- | --- | --- |
| Underlying disorder known to be associated with DIC | Required | Required |
| SIRS score | - | 0-2: 0 points  ≥3: 1 point |
| Platelet count | >10 x 10^4^/μL: 0 points  ≤10 x 10^4^/μL: 1 point  ≤5 x 10^4^/μL: 2 points | ≥12 x 10^4^/μL: 0 points  8-12 x 10^4^/μL (or >30% decrease/24 hrs): 1 point  <8 x 10^4^/μL (or >50% decrease/24 hrs): 3 points |
| Prothrombin time | <3 sec: 0 points  3-6 sec: 1 point  ≥6 sec: 2 points | Prothrombin time ratio:  <1.2: 0 points  ≥1.2: 1 point |
| Fibrinogen | >100 mg/dL: 0 points  ≤100 mg/dL: 1 point | - |
| Fibrin/fibrinogen degradation products | No increase: 0 points  Moderate increase: 2 points  Marked increase: 3 points | <10 μg/mL: 0 points  10-25 μg/mL: 1 point  ≥25 μg/mL: 3 points |
| Total DIC score | No DIC: <5 points  DIC: ≥5 points | No DIC: <4 points  DIC: ≥4 points |
| ISTH International Society on Thrombosis and Haemostasis, JAAM Japanese Association for Acute Medicine, DIC disseminated intravascular coagulation, SIRS systemic inflammatory response syndrome | | |
